# Assessment of dentists’ knowledge and awareness of oral manifestations of COVID-19 and the impact of pandemic waves on dental practice in India - an embedded study

**DOI:** 10.1186/s13104-025-07367-0

**Published:** 2025-07-31

**Authors:** Swati Bhatnagar, Shushma Rao B., Rahul Srivastava, Niharicka Gopalakrishnan, Ramya Shenoy, Roma Mascarenhas

**Affiliations:** 1 Manipal College of Dental Sciences Mangalore, Manipal Academy of Higher Education, Manipal, 576014 Karnataka India; 2 Department of Public Health Dentistry, Manipal College of Dental Sciences Mangalore, Manipal Academy of Higher Education, Manipal, 576014 Karnataka India; 3 M Public Health, University of Adelaide, Adelaide, South Australia; 4 Department of Public Health Dentistry, Manipal College of Dental Sciences Mangalore, Manipal Academy of Higher Education, Manipal, 576014 Karnataka India; 5 Department of Conservative Dentistry and Endodontics, Manipal College of Dental Sciences Mangalore, Manipal Academy of Higher Education, Manipal, 576014 Karnataka India

**Keywords:** COVID-19, Embedded study, Dentist, Awareness, Knowledge, Oral health, Health, Well-being

## Abstract

**Background:**

The cross-infection created between dental staff and patients by COVID-19 had no established protocols for the dental problems caused by the pandemic. Hence, the dentists’ knowledge regarding the oral symptoms of COVID-19 were assessed.

**Objective:**

This study reviewed the knowledge regarding oral manifestations of COVID-19 among dentists and the effect of waves 1 and 2 on dental community.

**Methods:**

In phase I, a cross-sectional survey with standardised and validated questionnaire was distributed among dentists using social media. In phase II, a qualitative in-depth interview of 8 dental clinicians (samples due to data saturation) was done to investigate the effects waves 1 and 2 on dentistry. Descriptive data was analysed via chi-square tests and Phase II analysis was carried out via Atlas-Ti software.

**Results:**

About 84.4% dental surgeons, 15 (3.7%) BDS, 48 (11.9%) MDS aspirants had good knowledge about the basic details and the oral manifestations of COVID-19, and the results were statistically significant with the participants’ knowledge about COVID-19 (*p* = 0.001), oral manifestations (*p* = 0), recommended personal prevention (*p* = 0.01), the necessity of RT‒PCR (*p* = 0) and disease fatality (*p* = 0.015). In phase II, four themes were formed from interviews: Source of 1st information, Outlook towards COVID-19, Impact and Self-retrospection.

**Conclusion:**

This study highlights the knowledge assessment among dental clinicians on the requirement of educational training programs about infection control practices to be followed in dentistry. The in-depth interviews of the dental clinicians revealed the need for strict infection control protocols following the pandemic.

**Supplementary Information:**

The online version contains supplementary material available at 10.1186/s13104-025-07367-0.

## Introduction

Global health and economic crises were caused by the coronavirus disease 2019 (COVID-19), which spread quickly and became a pandemic. Unlike SARS-CoV and MERS-CoV, this new virus is different, and its most likely source is Chinese horseshoe bats [1]. A member of the family Coronaviridae of the order Nidovirales, the causative organism causing this pandemic is severe acute respiratory syndrome coronavirus 2 (SARS-CoV-2) [2]. The primary means of genome transmission for this virus are droplet dissemination and contact pathways. Structurally, it is composed of massive, single-stranded RNA [3]. Although sputum production, headaches, haemoptysis, and diarrhoea are relatively rare symptoms, the main clinical signs and symptoms of this disease in humans include fever, cough, abnormal chest computed tomography (CT) images, and severe respiratory distress [3]. Researchers have confirmed the nosocomial transmission of SARS-CoV-2, although very little is known about its mode of transmission and extent of environmental contamination [4, 5, 6, 7]. This scenario has become more challenging because of reports of a substantial rise in the total number of silent carriers. Considering such circumstances, the first line of defence is to maintain a safe distance and refrain from making personal contact. By implementing the precautions periodically recommended by the WHO, the goal is to interrupt the chain of virus transmission [7]. In a dental setting, as the dentist and their equipment are near the patient, the chance of acquiring infection from the microdroplets of an infected patient is high, and owing to the characteristics of dental settings, the risk of cross-infection between dental health care personnel (DHCP) and patients can be very high [7, 8]. Dentistry has had to adapt to new epidemic circumstances to provide relief to patients in pain and prevent them from being a potential source of SARS-CoV-2 transmission [9].

In both developed and developing nations, dental problems are increasingly ranking among the most significant problems. Maintaining a clean oral cavity and improved systemic health reduces the development and spread of disease. To preserve this, one needs to be mindful of oral hygiene practices to preserve oral wellness as well as personal hygiene. Gaining knowledge about dental health is essential to enhancing one’s overall and oral health. The Indian dental health profession’s main objective is to increase consciousness and disseminate knowledge regarding oral hygiene practices [8]. Nondental care professionals are extremely concerned about the importance of oral hygiene during the COVID-19 pandemic, and it is the duty of the dental care specialists for raising awareness and educating pertinent group members. Infection control being an important aspect in dentistry, special care must be taken to protect patients, dentists, and other related parties from COVID-19 during the pandemic. It is also possible to discuss the difficulties encountered in dental practice and in obtaining oral health treatments, in addition to the financial ramifications brought about by Covid-19 and its effects on dentistry and oral health in general.

The purpose of this present study was to assess the knowledge and awareness with respect to oral manifestations of COVID-19 among the dentists nationwide and to evaluate the implications of both waves 1 and 2 of the pandemic for the dental community.

## Aim and objective

This integrated study aimed to assess dentists’ knowledge and awareness of the oral manifestations of COVID-19 and the impact of the first and second waves of the virus on dentistry in India.

## Main text

### Materials and methods

This embedded study followed two phases. In Phase I, a pre-made questionnaire based on existing scientific literature was formulated by three authors. Three dentists constructed the questionnaire’s validity and content analyses. External validation of the questionnaire was done by two BDS and MDS graduates. Following a validity check, three dentists assessed the questionnaire’s overall validity, and an agreement score was determined (Kappa value = 0.78). The authenticated survey was shared with dental professionals via various social media sites, including Facebook, Instagram, and WhatsApp (https://forms.gle/MfP8JDoHPUw4vRS88). The objective of Phase I of the study was to evaluate the knowledge and awareness among the dental clinicians Pan India about oral signs of COVID-19. The cross-sectional questionnaire included the basic knowledge about COVID-19, mode of transmission, awareness regarding the oral manifestations due to COVID-19, it’s manifestations, and precautionary measures. This study was approved and commenced by the Institutional Ethical Committee of the University bearing the IEC number 21,027. The targeted participants contained all dentists employed by the public and private sectors as well as academic institutions. Anonymity was preserved throughout the investigation. The Google form was shared with 480 participants; 404 (84.2%) academic and licensed dentists answered the surveys whereas 76(15.2%) were non-responders.

$$\:n=\frac{{\left[{Z}_{a}\right]}^{2}}{2d}$$

n = sample size.

Z_α =_ value at a specified confidence level.

Proportion of participants with knowledge of COVID-19.

d = acceptable margin of error (10%).

In Phase II, in-depth interviews with dental clinicians were conducted to investigate the effects of the pandemic’s first and second waves on dentistry. Being a qualitative study, in this phase, sample size was not determined. Codes, subcategories, and categories were created following each in-depth interview, and data saturation was assumed. The target population for this phase included BDS and MDS dental professionals with full-fledged clinical practice and dental consultants. The participants were approached for an open-ended interview by the primary investigator. With the help of an interview guide, the primary investigator conducted the interviews in a predesignated room where only the investigator and the participant were present. The interview guide was crafted through a synthesis of literature and insights provided by two researchers actively involved in the program since its inception. These researchers possessed first-hand experience in delivering clinical care to local inhabitants and were adept at conducting interviews. The guide comprised of open-ended inquiries aimed at eliciting comprehensive insights into participants’ perspectives. To ensure the validity of both face and content, three subject experts scrutinised the guide, followed by pilot testing on two adult patients not involved in the study, aimed at enhancing clarity and comprehensibility. The interview durations ranged between 15 and 25 min. These interviews were recorded via a digital voice recorder by a designated note-taker, with the resulting files securely stored in password-protected systems. Prior to the interviews, both oral and written informed consent was obtained, ensuring their anonymity to foster candid responses and mitigate social desirability biases. Following each session, the investigator synthesised key discussion points. No repeat interviews were conducted.

### Statistical analysis

Phase I, descriptive statistics was analysed with the help of Statistical Package for Social Sciences (SPSS), version 17 (SPSS Inc., Chicago, IL). Chi-square tests were applied for quantitative data. In phase II, the qualitative data was collected from the in-depth interviews and the field notes were transcribed verbatim. The transcripts were read and re-read by two investigators to gain a complete understanding of the interview content, highlighting the keywords. The Atlas-Ti software version 23 for Windows was used for coding and arriving at themes for the qualitative data. The data were labelled and coded. The codes were merged into categories, and various themes were identified. All the research team members who participated in the data analyses were referred to the participants only by their assigned codes. These identification codes were subsequently replaced with pseudonyms. All the data, fieldnotes, and transcriptions were kept in password-protected computers accessible only to the research members of the study.

## Results

### Phase I

The responses for the questionnaire survey were recorded from 404 dentists Pan India who consented to take part in the survey. From our scrutinization, 177 were males and 226 were female participants. The mean age of the study participants was 34.2 ± 9 years. The surveyed participants were grouped into 341 (84.4%) dental surgeons, 15 (3.7%) undergraduate students, 31 (7.7%) postgraduate students and 17 (4.2%) faculty. Based on the years of experience, a total of 51.7% (209) participants had < 5 years of experience, 27.7% (112) had 5–10 years, 13.9% (56) had 11–15 years, and 6.7% (27) had > 15 years of experience. All comparisons were performed based on the designation of the participants. All the responses of Phase I are grouped in Table 1.

**Table 1:** Frequency for each question based on the designation of the participants and the results of chi-square analysis

			General dentist *N* (%)	UG *N* (%)	PG *N* (%)	Faculty *N* (%)	Chi-square significance *p*
What is covid-19?		Pulmonary disease	2 (0.6%)	2 (13.3%)	2 (6.5%)	1 (5.9%)	**0.001**
		Immunity disorder	18 (5.3%)	-	-	1 (5.9%)	
		SARS-CoV-2 infection	318 (93.3%)	13 (86.7%)	29 (93.5%)	14 (82.4%)	
		Acquired zoonotic infection	3 (0.9%)	-	-	1 (5.9%)	
What is the incubation period?		2–14 days	302 (88.6%)	13 (86.7%)	29 (93.5%)	16 (94.1%)	0.74
		14–28 days	39 (11.4%)	2 (13.3%)	2 (6.5%)	1 (5.9%)	
Where was covid-19 first diagnosed?		China	335 (98.5%)	15 (100%)	30 (96.8%)	17 (100%)	0.44
		Italy	4 (1.2%)	-	-	-	
		USA	1 (0.3%)	-	1 (3.2%)	-	
What is the transmission route?		Aerosol transmission	32 (9.4%)	3 (20%)	5 (11.6%)	3 (17.6%)	**0.00**
		Droplet spread	11 (3.2%)	-	2 (6.5%)	-	
		Airborne	2 (0.6%)	-	4 (12.9%)	-	
		All	295 (86.8%)	12 (80%)	20 (64.5%)	14 (82.4%)	
What is the oral manifestation of covid-19?		Loss of taste	23 (6.7%)	5 (33.3%)	5 (16.1%)	5 (29.4%)	**0.00**
		Dry mouth & dryness and inflammation of the mouth	1 (0.3%)	1 (6.7%)	-	-	
		Mucormycosis	4 (1.2%)	-	1 (3.2%)	-	
		Ulcers and enlargement of lymph nodes	1 (0.3%)	-	-	1 (5.9%)	
		All the above	312 (91.5%)	9 (60%)	25 (80.6%)	11 (64.7%)	
Who are highly risked?		Nurses	5 (1.5%)	1 (6.7%)	-	-	**0.00**
		Dentists	217 (63.6%)	4 (26.7%)	9 (29%)	2 (11.8%)	
		Patients	-	-	-	-	
		All the above	119 (4.9%)	10 (66.7%)	22 (71%)	15 (88.2%)	
Can initial oral symptoms help in early detection?		Strongly agree	287 (84.2%)	6 (40%)	21 (67.7%)	12 (70.6%)	**0.00**
		Strongly disagree	3 (0.9%)	-	1 (3.2%)	2 (11.8%)	
		Not sure	51 (15%)	9 (60%)	9 (29%)	3 (17.6%)	
Which is the recommended personal protection to be followed?		Single masking	2 (0.6%)	-	-	-	**0.01**
		Double masking	2 (0.6%)	-	1 (3.2%)	-	
		Washing hands	1 (0.3%)	-	-	-	
		Maintaining social distance	-	-	-	1 (5.9%)	
		All the above	336 (98.5%)	15 (100%)	30 (96.8%)	16 (94.1%)	
Should RT‒PCR be done for every patient?		Strongly agree	233 (68.3%)	13 (86.7%)	20 (64.5%)	6 (35.3%)	**0.00**
		Strongly disagree	17 (5%)	-	3 (9.7%)	6 (5.3%)	
		Not sure	91 (26.7%)	2 (13.3%)	8 (25.8%)	5 (29.4%)	
Are you aware about	Availability of vaccine?	Yes	340 (99.7%)	15 (100%)	31 (100%)	17 (100%)	0.98
		No	1 (0.3%)	-	-	-	
	Method of diagnosing covid-19?	Yes	339 (99.4%)	15 (100%)	31 (100%)	17 (100%)	0.95
		No	2 (0.6%)	0	0	0	
	Fatality of disease?	Yes	340 (99.7%)	15 (100%)	31 (100%)	16 (94.1%)	**0.015**
		No	1 (0.3%)	-	-	1 (5.9%)	

UG– Undergraduate, PG– Postgraduate, N- Number of samples within the designation

Based on the responses received from the participants which assessed the degree of comprehension and knowledge, 93.5% of the respondents had a good knowledge about COVID-19 (SARS-CoV-2 infection). Chi-square analysis revealed a highly significant (*p* = 0.001) difference between the participants of various designations, indicating that COVID-19 was a SARS-CoV-2 infection. 89.1% of the participants provided the correct answer about the incubation of COVID-19 (2-14days). This demonstrates how crucial expertise and appropriate knowledge are to patient care. When asked about where COVID-19 got its initial diagnosis, 98.5% (397) of the subjects provided the correct response as China. There were no statistically significant results (*p* = 0.44) among the dentists, undergraduates and the faculty. 86.8% of the respondents mentioned COVID-19 spreads through aerosol, droplets and via air. A statistically significant result (*p* = 0.00) was obtained in the comparisons among the dentists with experience, calibre and designation for the response for mode of transmission of COVID-19.

91.5% of the participants responded about oral manifestations of COVID-19. They asserted that loss of taste, dry mouth, dryness, inflammation, mucormycosis, ulcers and enlargement of lymph nodes were manifested in COVID-19. Chi-square analysis showed highly significant difference (*p* = 0.00) compared with the designation and cadre of the dentists regarding the oral manifestations about COVID-19. 88.2% of the academicians asserted that nurses, dentists, and patients are always at risk. Chi square test showed highly significant results (*p* = 0.00) compared with the designation. This highlights the need for strict infection control strategies in the dental practice.

326 participants strongly agreed if initial oral symptoms help with early detection of COVID-19, whereas 6 participants strongly disagreed with this fact. 72 participants were not unsure of this fact. Chi-square analysis revealed highly significant results (*p* = 0.00) compared with the cadre of the dentists with knowledge of the diagnostic ability of initial oral symptoms. This highlights the need of special educational training programs (Table 1).

397 participants highlighted the need of personal protection as prime importance. The need of measures like single masking with N95, double masking, washing hands regularly and maintaining social distance of 2–3 feet were asserted. There were statistically significant results (*p* = 0.01) for the different designations. The need of personal protection barrier among the dental fraternity ensures the need of precise treatment strategies with stringent infection control measures.

272 respondents strongly agreed to the need of PCR as investigation procedure for every suspected individual. In the analysis with the chi-square test, there was a highly significant (*p* = 0.00) difference between the BDS and MDS aspirants. 99.8% participants were highly aware about the availability of vaccines as the government laid down the need of every individual to be vaccinated. 402 participants of different designations and cadre were aware of the method of diagnosing COVID-19 and there was no statistically significant difference (*p* = 0.95) as per chi-square analysis. 99.5% respondents were aware about the fatality of the and there was a statistically significant results (*p* = 0.015) in the comparisons for different designations (Table 1).

### Phase II:

In-depth interviews were conducted in the second phase of this study. Content saturation was achieved with 8 male participants. The primary investigator conducted the interviews via the interview guide. The coding of the interviews resulted in the identification of four primary themes: Source of 1st information, Outlook towards COVID-19, Impact and Self-retrospection. Three of the themes were further subdivided into two subthemes emerging from the respective codes. A comprehensive overview of these themes, subthemes, and codes is presented in Table 2 below.

**Table 2:** A comprehensive overview of these themes, subthemes, and codes

Themes	Sum-themes	Codes
Source of 1st Information	Media	News
		Social media
		Article
	Others	Newspaper
		Unsure source
Outlook towards covid-19		Positive
		Negative
Impact	Impact of Covid-19	Affected by the pandemic
		Unchanged circumstances
		Uncertain
	Impact according to the Covid-19 waves	First wave
		Second wave
Self-retrospection	Resources for facing pandemic	Well equipped
		Ill equipped
	Knowledge of pandemic	Improved understanding of disease

**Fig. 1:**
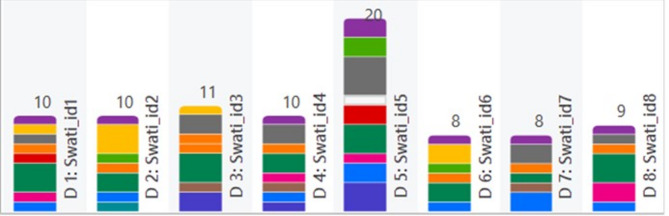
Coding of the participants’ interviews via software. Each bar represents a document from each participant

Each code was assigned a distinct colour to facilitate easy identification of the codes in the transcribed interviews. A depiction of the incidence of the codes in each participant interview is illustrated in a colour-coded chart, which is represented in Fig. 1. This helps in determining how prevalent each code is in all participant interviews. The ensuing segments offer a comprehensive discussion of every theme, supported by representative quotations (Table 3).

**Table 3:**
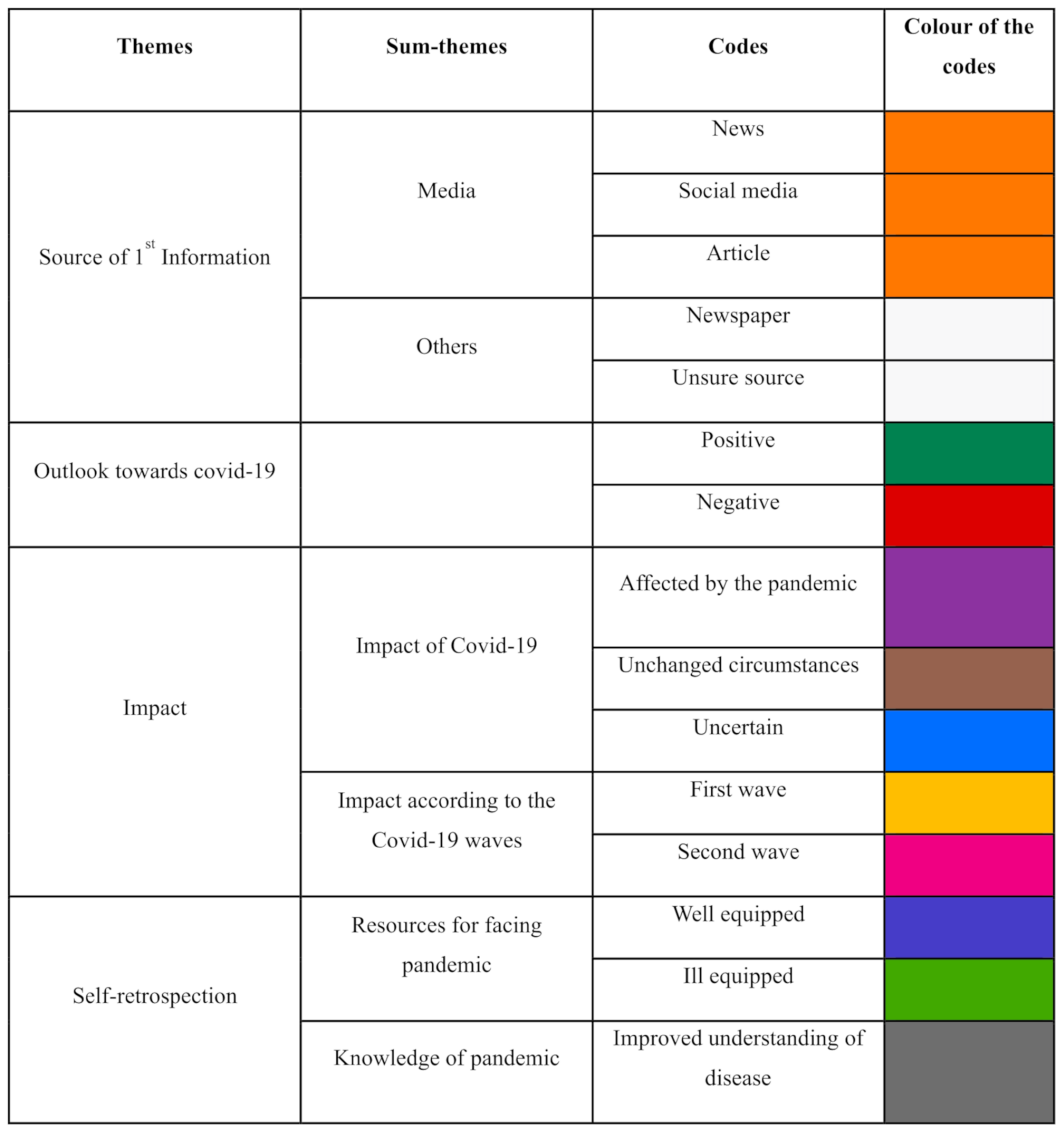
Representation of various themes according to colour coding

1. First theme: Source of 1st information: This theme was based on the source from which the dentist first received the news of the presence of COVID-19 and acquired information pertaining to it. Further this theme was subdivided into two subthemes: media and others. Most of the dentists (*n* = 6) said their source was media, i.e., from news, social media and articles, as depicted in the following excerpt:

*“My primary source of information was social media”– Id1*.

*“News articles*,* internet”– Id3*.

Two of the participants initially received information from other sources, one from a newspaper and the other from an unsure source. This is depicted in the excerpt below:

*“I got to know about it from a newspaper when I read that a new disease is emerging*,* then the news spreads in the media”– Id7*.

*“I am not exactly sure where I heard it first*,* maybe the news or somebody talking about it*,* I am not exactly sure”– Id8*.

2. Second theme: Outlook towards COVID-19: The theme was based on the experiences of practitioners during the pandemic and what they underwent as clinicians. These were either positive or negative in nature. Most of the responses were positive rather than negative. This is depicted in the excerpts below:

### Positives

*“Yes*,* I feel the recommended guidelines helped a lot*,* equally helped in both waves. I was feeling good at work”– Id1.*

*“Knowing the oral manifestations*,* I was fine with working as I knew what to look out for. Luckily*,* none of my patients came in with symptoms… They were honest about their health.”– Id3.*

### Negatives

*“I feel there was no control at all*,* few were following rules in dentistry as well as outside*,* as in people would lie about symptoms which made the whole period difficult for practitioners.”– Id2.*

*“Dentists were hit mainly because few dentists*,* as well as social media*,* together demonised dentistry*,* which created fear among the people.”– Id5.*

3. Third theme: Impact: This theme was derived from two subthemes, ‘**Impact of COVID-19’**, which had codes related to the overall impact of the pandemic, and **‘Impact according to the COVID-19 waves’** had codes if the participants were more prepared for the first or second wave.

- Impact of COVID-19: The participants emoted the effect of the pandemic on them and their dental practices. Some participants were affected by few circumstances, some participants were unaffected, and there was no change in their practice. A few excerpts are given below:

*“The first wave has had an impact on everyone’s career*,* but even then*,* it was not for long that it affected dentistry*,* maybe a little bit it did”- Id1.*

*“Since it was a sudden calamity*,* we dentists were definitely not prepared*,* and it did affect us.”– Id2.*

- Impact according to the COVID-19 wave: The participants were queried regarding the wave that posed challenges to the practice of dentistry. 50% agreed that it was the initial wave, whereas the remaining half perceived it to be the subsequent wave. Few responses are shown below.

*“In the first wave*,* the dentists were apprehensive*,* but in the second wave*,* since people were dying openly*,* people died in very large numbers*,* and hence*,* the second wave was more frightening”– Id5.*

*“The first wave has definitely affected the dentists and their career; people had to face long lockdowns and were truly scared of ant treatments*,* even dental treatment.”– Id6.*

4. Fourth Theme: Self-retrospection: The two subthemes under this theme were **‘resources for facing the pandemic’** and **‘knowledge of the pandemic’**. These subthemes were based on participants’ self-experience as dentists and practitioners during the pandemic. Few excerpts are depicted below.

- Resources for facing pandemics:

*“Once we got to know what precautions needed to be taken*,* I think everything worked. We just had to modify our practice a bit*,* to protect all around us*,* get few pieces of equipment and we were set for regular practice”– Id4.*

*“In the first wave*,* practitioners went for a long time without knowing how to manage practice considering the high risk of spread. There was not much idea of knowing who was a carrier too*,* took long to manoeuvre my way back to regular practice”– Id– 2.*

- Knowledge of the pandemic:

*“I feel the newcomers to practice might have found it difficult than us as they were just learning the tricks of the trade*,* but then there was good number of clear guidelines and rules which were given. This made it easier for all to know how to manage everything.”– Id7.*

## Discussion

The COVID-19 pandemic was a special time characterised by several socio health-economic problems. In addition to the general population, it has impacted dentistry and health care providers. Hospital staff who care COVID-19-positive patients either directly or through indirect contact have been greatly impacted. The potential danger of infection is extremely significant for dental fraternity. Patients’ blood and saliva are directly in contact with the dentists as they treat the oral cavity [2]. Even though dentists follow general precautions, COVID-19 has raised concerns about the necessity for extra precautions to prevent infection. Airborne particles are produced during dental operations such as restorative treatments, oral prophylaxis, and with the use of air rotar handpiece. Aerosol creation and distribution cannot be stopped, even with PPE kits, and can result in infection [4].

Dentists have benefited somewhat from WHO and IDA recommendations against COVID-19 prevention, but a stress-free workplace is still a ways off. An acceptable degree of COVID-19-related knowledge must be present among affected communities to address the issues and set realistic expectations regarding the disease’s future trajectory [2]. It is crucial to have a comprehensive understanding of the dissemination, mode of spread, early detection, and prevention of SARS-CoV-2 [5]. Therefore, the goal of the current study is to assess dentists’ awareness, and knowledge regarding COVID-19 infection which followed embedded study design.

An embedded research design is a mixed-methods analysis technique that collects both qualitative and quantitative data and incorporates the qualitative findings into the quantitative data. Thus, the study followed an embedded design in which the qualitative and quantitative data were merged. There are only a handful of studies following this methodology. Phase 2 was based on a qualitative study design wherein the sample was achieved based on content saturation, which was deduced when the codes and themes generated after each interview followed a recurring pattern. In the present study, this was achieved at 8 participants, which resulted in this sample size [4].

There were 404 participants in the first phase of this study, which was carried out to assess the knowledge and awareness of the dentists regarding COVID-19. Each participant in this study had a fair level of awareness, knowledge, and attitudes regarding oral health and maintenance care throughout the 2020 COVID-19 pandemic. This was in accordance with studies conducted by Priyadeep Banerjee et al. [10]

Most of the participants were aware that COVID-19 was a severe acute respiratory syndrome coronavirus 2 (SARS-CoV-2) infection and presented significant results, with the highest percentage of general dentists responding the same way. These results are in line with the studies of Pasupuleti MK et al. [5]. In a study by Kumar et al., the understanding of dental health care professionals about COVID-19 was mediocre and concluded that educational training programmes should be conducted to improve the knowledge [2]. The degree of knowledge of the respondents in this study was fair when compared to studies by Roy et al., Abbas NQ et al. [11, 12]. Nonetheless, they demonstrated a positive outlook and a respectable standard of practice because they followed directives from many national and international health organisations. Similar results were also noted in studies by Eby Varghese et al. [6]. Various studies support that COVID-19 is a severe acute respiratory syndrome coronavirus 2 (SARS-CoV-2) infection that has spread throughout the world and is responsible for millions of deaths [3, 8, 12]. The government of India, in its FAQ release, asserted that the incubation period for COVID-19 is up to 14 days, which was known by most of the participants in this study which were coinciding with the results present in Kumar et al. [2, 8, 11]. Medical personnel can use this data to determine if isolating a person who has come into proximity to the virus is necessary [9].

Most of the participants agreed that the first diagnosed case was in China, and many researchers have affirmed this fact in their works [2, 8, 9, 10, 11, 12, 13, 14, 15]. The route of transmission for COVID-19 was determined by most of the participants as being spread through droplets, aerosols and airborne transmission, and similar results were reported in a study by Teja et al. [17]. These transmission pathways result in the propagation of COVID-19 when it originates from the expirations of a COVID-19 patient [16]. The most common oral sign that has been reported is loss of taste [17]. There are several theories detailing how COVID-19 might have been connected with ageusia [18, 19, 20]. According to one of these theories, rhinitis may impair normal taste bud function by causing corresponding localised inflammation [20]. Another hypothesis for ageusia could be the side effects of the drugs consumed by patients for COVID-19 [18]. According to research by Veira RA et al., COVID-19 patients have multiple ulcers. They identified anxiety as a contributing element for these ulcers [21]. Works from several researchers and this study revealed that several oral symptoms, such as dysgeusia, ageusia, ulcers, lymph node enlargement, xerostomia, and mucormycosis, are present in COVID-19 patients and can be very beneficial in the early diagnosis of this disease [22, 23, 24]. Studies by Natto et al. and Gluckman et al. revealed that dentists and nurses are highly at-risk professionals for becoming infected by COVID-19 owing to their close proximity to patients and their respiratory and oral aerosols [13, 25–26]. Most of the participants agreed that the oral manifestations of COVID-19 can help in the diagnosis of the disease, as agreed with the study by Wolf et al. [27].

As per the guidelines of the government, frequent hand washing and the use of masks help prevent the acquisition and spread of COVID-19 [10]. Similar results were reported in this study and another study conducted in Saudi Arabia [2].

The dentists in the present study agreed that all the above measures are necessary to keep COVID-19 at bay which coincide with other studies [28, 29]. Although the WHO does not recommend that every patient receive an RT‒PCR test, it is mandatory prior to undergoing any surgical procedure that can generate many aerosols [26, 27]. However, in this study, many of the participants agreed that all patients needed RT‒PCR, indicating that they were over precautious regarding the spread of COVID‒19.

In phase II, in-depth interviews were conducted with the participants who had private practices to obtain their views on COVID-19 and its impact on dental practice. The responses were divided into four themes: source of first information, outlook towards COVID-19, impact and self-retrospection, each comprising various codes. The participants had gained their first knowledge of COVID-19 either through media (news, social media, and articles) or through other sources, such as newspapers. The responses were similar in studies reported in Saudi Arabia and Spain [2, 30]. The outlook towards COVID-19 was grouped into positive or negative outlooks of the participants regarding their dental practices during COVID-19. There were more positive responses where dentists affirmed that, owing to following the guidelines, there was no difficulty in continuing their practice. The negative responses were like those reported in a study conducted by Parvin et al., where fear was one of the main factors hindering dentistry [31].

The impact theme was divided into subthemes of dentistry being affected overall by COVID-19 and affect based on the first and second waves. The participants reported that there was a significant impact on their career trajectory, albeit transient, indicating a short-term effect on their dental practice. Similar reports have been recorded [31]. This impact could be the result of COVID-19 being highly prone to transmission through several aerosol-generating procedures, and dentistry has its own basis on these procedures [32].

The theme of Self-retrospection was based on the experience of the participants and included two subthemes: resources for facing the pandemic and knowledge of the pandemic. This theme spoke of how equipped the participants were to face the pandemic and their understanding of COVID-19. Despite having good knowledge regarding the precautions to be taken, dentists agreed that there were challenges in the initial period where the protocol was not clear. A lack of guidelines was also reported in a study conducted in Jordan [33], and Hisn Chung et al. reported an insufficient protocol for dental practice [34, 35, 36].

The study presented with certain limitations. First, Phase I of this study was carried out through an online questionnaire, which may have resulted in reduced accuracy of responses from the participants compared with direct interviews. However, the high sample size (*n* = 404) ensures the validity of the responses. Second, phase II had fewer participants, which is a known drawback of a qualitative study, and future studies may be conducted to ensure greater participation of dentists.

## Conclusion

This study highlights respondents’ overall knowledge was fair about the oral manifestations of COVID-19 among the dental personnel in India. Although there is a sufficient understanding of the issue, there is room for development. Competent information, an optimistic outlook, and scientifically proven procedures are necessary for the effective control of any outbreak. Dentistry curricula should involve the addition of appropriate protocols of infection control practices for the prevention. Clinicians and nondental professionals must thrive to obtain additional information with new norms of infection control practices through research, ongoing dental education, healthcare training, and symposiums.

## Electronic supplementary material

Below is the link to the electronic supplementary material.

Supplementary Material 1

## Data Availability

No datasets were generated or analysed during the current study.

## Abbreviations

COVID-19: Coronavirus disease 2019
SARS-CoV-2: Severe acute respiratory syndrome coronavirus − 2
MERS-CoV: MERS-related coronavirus
WHO: World health organization
IDA: Indian dental association
