# Supplementary material for: Assessment of dentists’ knowledge and awareness of oral manifestations of COVID-19 and the impact of pandemic waves on dental practice in India - an embedded study
Source: BMC Res Notes. 2025 Jul 31;18:342. doi: 10.1186/s13104-025-07367-0 (PMC12315399; doi:10.1186/s13104-025-07367-0)
Supplement: Supplementary file 1 — Supplementary Material 1 [file 13104_2025_7367_MOESM1_ESM.pdf]

Menu

- English
  中文
  日本語
  English
  Português
  Español

- **AJE**
  - My Orders
  - My Invoices
  - Refer a Colleague
  - My AJE Rewards
  - My Prepay and Credits

  Rubriq
  - Workspace
  - My Plan

  My Account
  - Settings
  - Log out

Authoring Services

- English Editing
- Academic Translation
- Scientific Editing
- Journal Recommendation

Formatting Services

- Manuscript Formatting

Funding Services

- Grant Services

Automated Tools

- Grammar Check

Rubriq

- About & Pricing

Learn

- Author Resources
- Help Center
- Legal

Company

- About AJE
- Careers
- What Sets us Apart

- Pricing Get a Price Quote
- Order now

- AJE Logo
- Services

Authoring Services

- English Editing
- Academic Translation
- Scientific Editing
- Journal Recommendation

Get a Price Quote Contact Us
All Services

Formatting Services

- Manuscript Formatting

Research Promotion

- Research Promotion

Funding Services

- Grant Services

Automated Tools

- Grammar Check
- Resources

Learn

- Author Resources
- Help Center
- Legal

Company

- About AJE
- Careers
- What Sets us Apart
- Pricing

- Rubriq New
- English
  中文
  日本語
  English
  Português
  Español
- My Account
  - AJE
  - My Orders
  - My Invoices
  - My AJE Rewards
  - My Prepay and Credits
  - Refer a Colleague
  - Rubriq
  - Workspace
  - My Plan
  - Account
  - Settings
  - Get Help
  - Log out
- Order now

- 中文 日本語 English Português Español

Welcome back, Amitha Lewis

AJE Grammar Check

Manuscript Swati 14-1-24 (1).docx

Evaluated on 2025-01-24
Back to recent evaluations · Archive

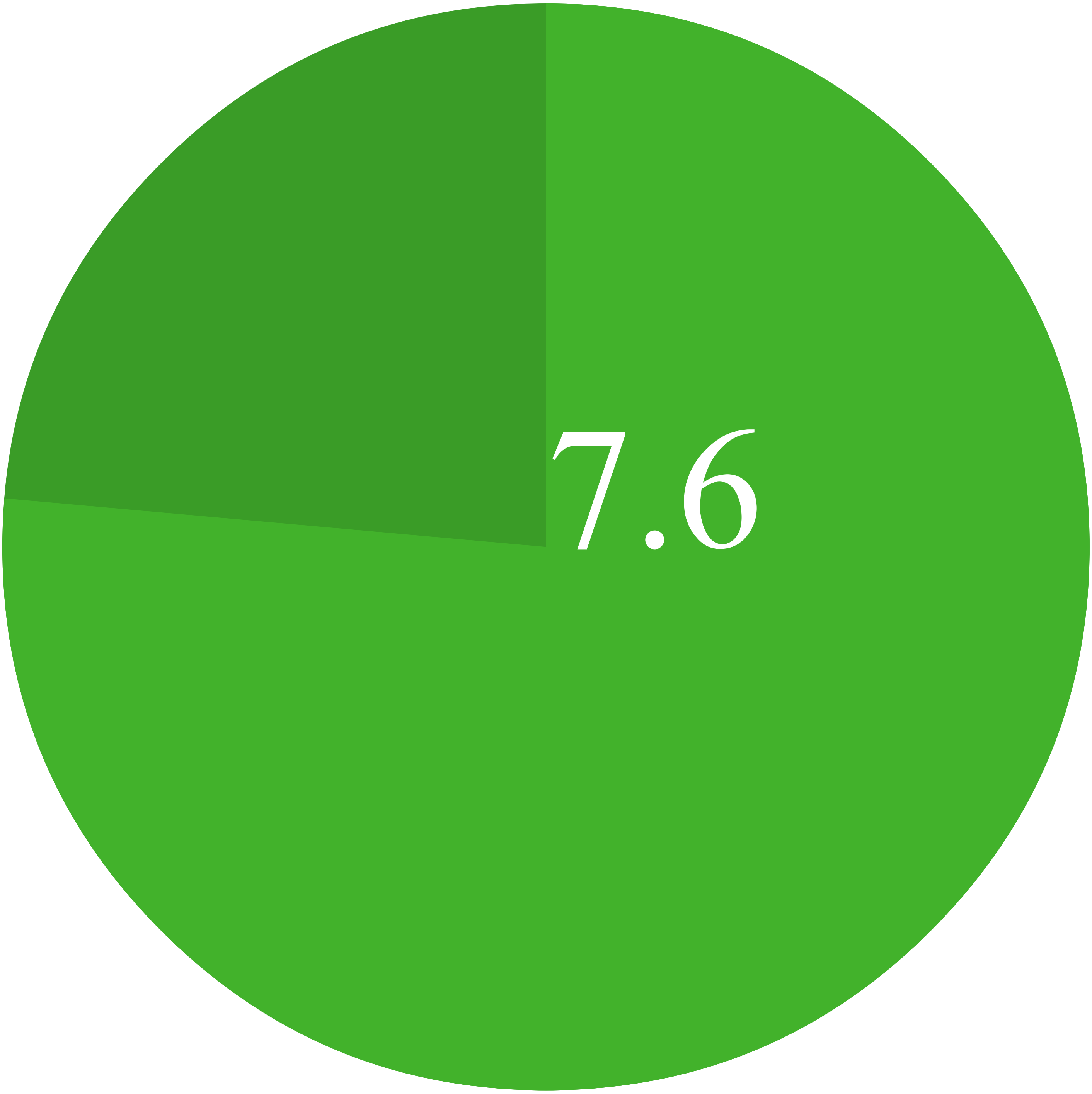

Your Language Assessment Score (LAT)

Your paper scored a **7.6/10**. This places it in the **89th** percentile of papers submitted to AJE for language assessment.

Invest in Success: On average, papers edited by AJE improve their score to 8.7 and move up to the top 5% (95th percentile) of research papers submitted to journals.

Ensure your polished research makes a lasting impact. AJE's expert editing can give your already strong paper that extra edge for high-impact journals.

■
Average language quality score after editing
●
Your current language quality score

Areas for Improvement

Our software classifies several different types of errors common in scientific and academic writing. We found 127 potential errors in this manuscript, shown in the graph to the right.

There are a total of **127** errors in your manuscript.

Elevate Your Research with AJE Author ServicesService Options

Presubmission Review

Get expert feedback on structure, presentation, logic, and communication of your research's relevance and impact. An editor in your field will provide in-line comments to help you prepare for peer review.

LEARN MORE

Journal Recommendation

Our team of academic experts will recommend 3 suitable journals for your article based on its topic, scope, and your specific goals (e.g., preferred Impact Factor range and index).

LEARN MORE

Manuscript Formatting

Ensure your paper adheres to your chosen journal's formatting guidelines. Our experts will modify references, citations, page layout, text formatting, headings, title page, and figure placement for a polished submission.

LEARN MORE

Our Team

Employee headshot

Ashley Smith, PhD

PhD, Epigenetics and Cell Biology
Brown University

"After graduating from Roger Williams University with a B.S. in Biology and and B.A. in Chemistry, I began working at Brown University studying nanotoxicology under Dr. Agnes Kane. It was at Brown University that I first became interested in epigenetics. After working as a research assistant for two years, I realized that I wanted to extend my education and applied to several doctoral programs focusing on epigentics and cell biology. I chose to continue my studies at Brown and, while there, I was lucky to collaborate with UCSF to research the correlation between genetic and epigenetic patterns in human glioma. Our goal was to identify methylation profiles in brain tumors and associate these patterns with histology, outcome, and immune response. I received my PhD from Brown in 2013."

Free Resources from AJE Scholar

Stuck on your introduction? Confused about paragraph structure? Need help crafting a compelling cover letter? AJE Scholar is your one-stop shop for free resources to elevate your academic writing at every stage.

Top 3 Reasons to Post a Preprint
Many researchers are choosing to post a preprint (a full draft of a research paper that hasn't yet been peer reviewed), before they submit to a journal. Here are the top 3 reasons why you should post your manuscript as a preprint.

Avoiding Image Fraud: 7 Rules for Editing Images
Figures represent an extremely important, yet often overlooked, aspect of a scientific paper. Learn how to use and adapt them without violating copyright.

Scientific data presentation: a picture is worth a thousand words
Graphs and tables are powerful storytelling tools and are critical components of scientific publications. Learn different ways to present data and things to consider

AJE Logo

AJE's comprehensive suite of high quality author services enable researchers to spend less time preparing their work for publication and more time doing the research that drives society forward. We achieve this by developing innovative software and high quality services for the global research community. Our team is

made up of researchers and industry professionals working together to solve the most critical problems facing scientific publishing.

Follow us on Twitter Join us on Facebook Follow us on Instagram Connect on Linkedin

About

- About AJE
- Services
- Pricing
- Contact Us

Resources

- AJE Scholar
- Ethics
- Quality
- AJE Rewards
- AJE Grammar Check

rubriq

- About & Pricing

Company

- Company
- Careers

Legal

- Security & Privacy
- Terms of Service
- Do Not Sell My Personal Information

© 2025 American Journal Experts - All rights reserved.
